# A116 PREVALENCE OF EDITORIAL BOARD MEMBERSHIP AMONG ORIGINAL RESEARCH PUBLISHED IN HIGH IMPACT GASTROENTEROLOGY-FOCUSED JOURNALS: A CROSS-SECTIONAL ANALYSIS

**DOI:** 10.1093/jcag/gwad061.116

**Published:** 2024-02-14

**Authors:** Y Verma, M Scaffidi, N Gimpaya, A Li, R Khan, S C Grover

**Affiliations:** University of Toronto Temerty Faculty of Medicine, Toronto, ON, Canada; Medicine, Queen's University Faculty of Health Sciences, Kingston, ON, Canada; Division of Gastroenterology, St. Michael's Hospital, Toronto, ON, Canada; Division of Gastroenterology, St. Michael's Hospital, Toronto, ON, Canada; Division of Gastroenterology, St. Michael's Hospital, Toronto, ON, Canada; University of Toronto Temerty Faculty of Medicine, Toronto, ON, Canada

## Abstract

**Background:**

Editorial board members, as experts in their field, often publish relevant research in journals on which they serve. This combined role of “editor-as-author” may be a source for conflict of interest in the peer review process. In Gastroenterology-focused journals, the prevalence of instances for editor-as-author has not been explored.

**Aims:**

To estimate the prevalence of editor-as-author among high impact factor (IF) Gastroenterology-focused journals.

**Methods:**

We used a cross-sectional study design. We used InCites Journal reports to identify 30 Gastroenterology journals with the highest IF in 2020, wherein we included journals that had publicly accessible board membership that indicated the following: institution/city/country; and at least the first name initial and entire last name for editorial board members. We created a list of all editorial board members, excluding any emeritus editors and society representatives. Among these journals, we identified all original research articles using the Web of Science, wherein we excluded any editorial material, meeting abstracts, and clinical practice guidelines. Among these original publications, two authors then independently and in duplicate determined any instances of authors who had editorial board membership. The primary outcome measure was the prevalence of editors-as-authors. Secondary outcomes involved the number of editorial board members with first authorships and senior/corresponding authorships, as well as the number of articles that disclosed editorial board membership.

**Results:**

Among the 30 Gastroenterology-focused journals that had the highest IF, 24 met the relevant inclusion criteria. These journals had a total of 6162 original publications, of which 992 (16.1%) had at least one instance of editor-as-author; 8 (0.1%) articles disclosed that there was editorial board membership among its authors. Among all 24 included journals, there were a total 2106 editorial board members. Of these members, 635 (30.2%) had at least one instance of editor-as-author, wherein 134 and 263 members were first authors and senior/corresponding authors, respectively.

**Conclusions:**

Our study found that approximately one third of editorial board members among high IF Gastroenterology-focused journals were also authors in these same journals. Future analysis will focus on determining whether there is the difference in quality of published work compared to those without editorial board membership.

**Funding Agencies:**

None

